# A new fusion protein platform for quantitatively measuring activity of multiple proteases

**DOI:** 10.1186/1475-2859-13-44

**Published:** 2014-03-21

**Authors:** Chengdong Zhou, Yanping Yan, Jie Fang, Beijiu Cheng, Jun Fan

**Affiliations:** 1 Key Laboratory of Crop Biology of Anhui Province, Anhui Agricultural University, 130#, Changjiang West Road, Hefei City, Anhui Province 230036, PR. China

**Keywords:** Diaminopropionate ammonia-lyase, Fusion proteins, *E. coli*, Production, Coupled assay, Specific proteases

## Abstract

**Background:**

Recombinant proteins fused with specific cleavage sequences are widely used as substrate for quantitatively analyzing the activity of proteases. Here we propose a new fusion platform for multiple proteases, by using diaminopropionate ammonia-lyase (DAL) as the fusion protein. It was based on the finding that a fused His6-tag could significantly decreases the activities of DAL from *E. coli* (eDAL) and *Salmonella typhimurium* (sDAL). Previously, we have shown that His6GST-tagged eDAL could be used to determine the activity of tobacco etch virus protease (TEVp) under different temperatures or in the denaturant at different concentrations. In this report, we will assay different tags and cleavage sequences on DAL for expressing yield in *E. coli*, stability of the fused proteins and performance of substrate of other common proteases.

**Results:**

We tested seven different protease cleavage sequences (rhinovirus 3C, TEV protease, factor Xa, Ssp DnaB intein, Sce VMA1 intein, thrombin and enterokinase), three different tags (His6, GST, CBD and MBP) and two different DALs (eDAL and sDAL), for their performance as substrate to the seven corresponding proteases. Among them, we found four active DAL-fusion substrates suitable for TEVp, factor Xa, thrombin and DnaB intein. Enterokinase cleaved eDAL at undesired positions and did not process sDAL. Substitution of GST with MBP increase the expression level of the fused eDAL and this fusion protein was suitable as a substrate for analyzing activity of rhinovirus 3C. We demonstrated that SUMO protease Ulp1 with a N-terminal His6-tag or MBP tag displayed different activity using the designed His6SUMO-eDAL as substrate. Finally, owing to the high level of the DAL-fusion protein in *E. coli*, these protein substrates can also be detected directly from the crude extract.

**Conclusion:**

The results show that our designed DAL-fusion proteins can be used to quantify the activities of both sequence- and conformational-specific proteases, with sufficient substrate specificity.

## Backgound

Proteases play key roles in many fundamental cellular, viral processes and widely used as tools in biotechnology [1,2]. One major usage of proteases is to cleave off fusion tags from target proteins, which are either used as solubility tag and for affinity tag. The solubility tags, for example, small ubiquitin-related modifier (SUMO) tag, have a promoting effect on the expression and folding of the target protein. The affinity tags such as hexa-histidine tag (His6-tag) and chitin binding domain (CBD) facilitate rapid purification of the fusion protein. Others tags including maltose-binding protein (MBP) and glutathione S-transferase (GST) have both solubility and affinity benefits [3]. After purification, these fused tags are usually required to be removed, which otherwise would often inhibit the target protein activity, affect protein crystallization and cause the undesirable immune response for proteins in human therapy [3-6].

Several specific proteases have been used for this propose. Among them, TEVp protease (TEVp), rhinovirus 3C protease (R3P), thrombin, factor Xa, enterokinase is sequence-specific [4]. SUMO protease Ulp1 is dependent on the conformation of SUMO [5]. Some inteins and protease are processed by inducible self-cleavage [6]. To remove the fused tag, a linker sequence between the tag and target protein is used as the site for specific protease recognition and cleavage. Similarly, this type of fusion protein can also be used as substrate to measure the activity of protease for developing more potent and specific proteases, such as HIV protease and caspase-3 [7].

In the past, different types of protease substrates had been developed. One is using small molecule fluorescence probes. These probes are highly sensitive and can be used to monitor the cleavage kinetics of the target protease [8]. Another approach is to use the fluorescence resonance energy transfer construct between two fluorescent protein conjugates. This method has been wildly applied in the development of highly sensitive and specific protease activity assays in vivo and in vitro [9-11]. However, these synthetic probes are generally expensive. On the other hand, fusion protein substrates produced in *E. coli* are more cost-effective, and several of them had been tested for analyzing the sequence-specific proteases *in vitro*[12-14], or *in vivo*[15,16], or both [7]. Another fusion protein substrate has also been developed for *in vitro* analyzing SUMO-specific protease [17]. However, assays of these designed fusion protein substrates are inconvenient, because separation of the cleaved and uncleaved products is required to quantify the cleavage [12,13,18-20]. In addition, the ability of several protein substrates is further limited by their low stability under relatively high temperature or in the presence of denaturant [7,12-14]. Thus, developing new fusion protein substrate that has better stability and can be directly assayed is of great interest.

Diaminopropionate ammonia-lyase (DAL, EC 4.3.1.15), is a member of the fold type II family of pyridoxal 5’ phosphate (PLP) dependent enzymes. DAL catalyzes α, β-elimination reaction of L- or D-α, β- Diaminopropionate (L-DAP and D-DAP) to form pyruvate and ammonia [21]. It was shown that a N-terminal His6-tag significantly could decrease the *E. coli* DAL (eDAL) or *S. typhimurium* DAL (sDAL) activity [21]. Based on this finding, our previous study demonstrated that the activity of His6GST fused eDAL (GST-eDAL) was also decreased and could be rescued dramatically (~10-fold) after TEV cleavage of the His6GST-tag. Since the cleaved product (eDAL) can be directly assayed without further separation, His6GST-eDAL allows quantitative analysis of TEV protease (TEVp) variants activity in vitro and within *E. coli* cells [22]. Other tests also indicated that this fusion protein substrate can be used for detecting TEVp stability at temperature 30-45°C or in the supplementary denaturant including urea or guanidine hydrochloride up to 2 M [23]. Here, we broadened the application of the fused DAL (DAL-fusion) as substrate for other specific proteases. First, by combining different fused tags and the protease cleavage sequences, we selected the top DAL-fusion constructs with soluble expression of the fusion protein in *E. coli*. Then, we determined performance of the select DAL-fusion proteins as substrate for the corresponding proteases. From this systematic analysis, we developed four active DAL-fusion substrates suitable for TEVp, factor Xa, thrombin and DnaB intein, and summarized potential factors that may affect the development of other fusion protein substrates in the future.

## Results

### Design and overall results of the DAL-fusion substrate screen

To determine if DAL-fusion can be used as substrate for other proteases and to select the optimal fusion construct, we designed a library of DAL-fusion constructs, by combining seven different protease cleavage sequences (R3P, TEVp, factor Xa, Ssp DnaB intein, Sce VMA1 intein, thrombin and enterokinase), four different tags (His6, GST, CBD, MBP) and two different DALs (eDAL and sDAL), total of seventeen constructs. Among them, we successfully developed four active DAL-fusion substrates suitable for TEVp, factor Xa, thrombin and DnaB intein. In addition, our results also showed that substrate performance varied greatly with various factors. For example, incorporation of Sce VMA1 intein between CBD and eDAL caused the low expression level (see Additional file 1: Figure S1) and we did not further analyze the VMA1 intein activity. Similarly, no soluble expression level was obviously detected for CBD fused eDAL containing Ssp DnaB intein (data not shown), and replacement of CBD with MBP significantly enhanced production of the fusion protein. Also, substitution of GST with MBP increased the soluble yields of the R3P sequence fused eDAL. Although enterokinase cleaved eDAL at undesired positions, it did not process sDAL. We also demonstrated that SUMO protease Ulp1 with an N-terminal His6-tag or MBP tag displayed different activity using the designed His6SUMO-eDAL as a substrate. Highlights of the results are detailed as below.

### Effects of fused tag on the production and activity of eDAL or sDAL

**Figure 1:**
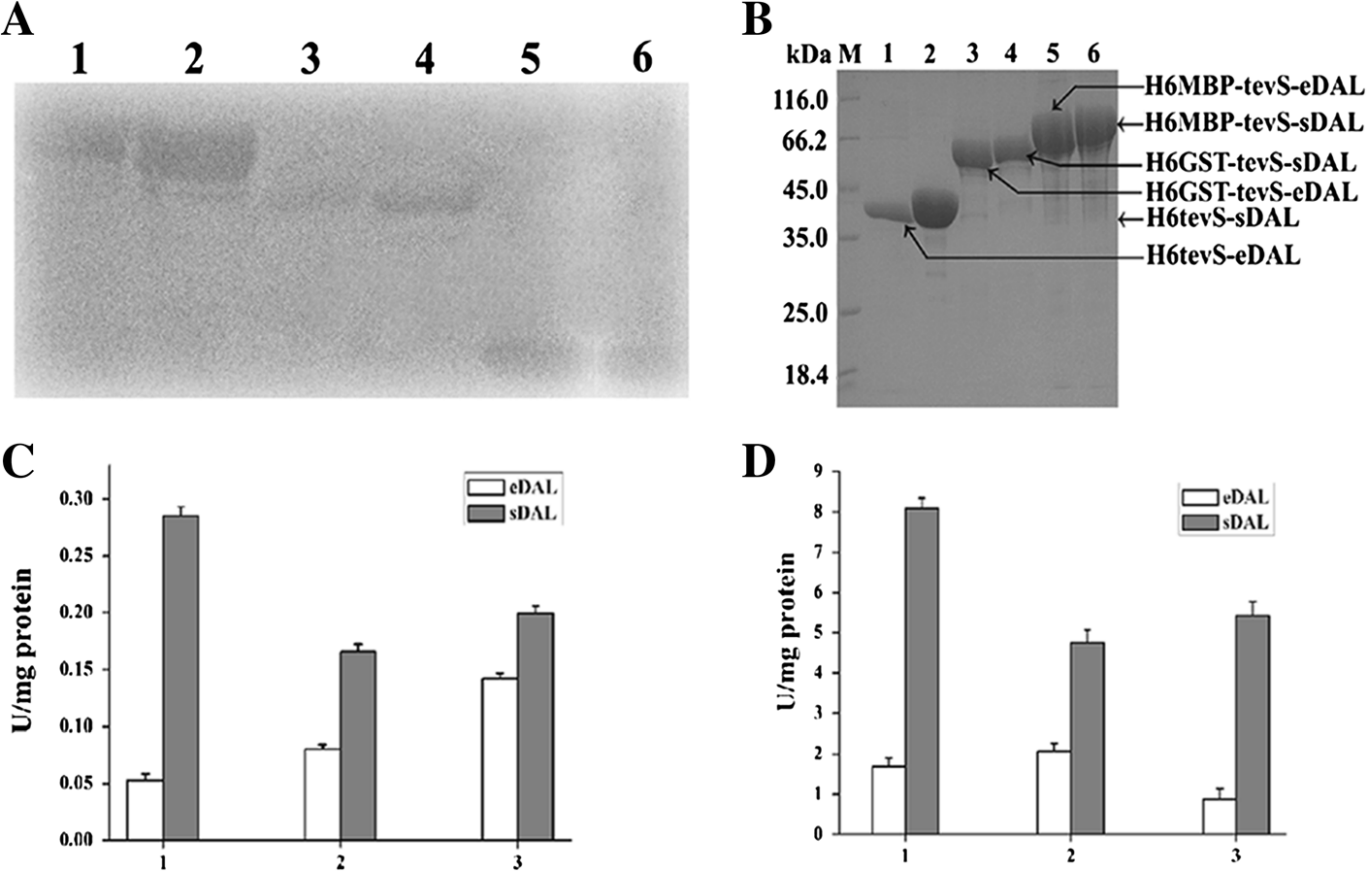
**Overexpression**, **purification and DAL activity assay of the fusion protein. A**. Expression of the soluble fusion protein for eDAL or sDAL with a specified tag followed by tevS in *E. coli*. The fusion proteins from the supernatants were detected by Western blot using anti-His6 antibody. The lane 1–6 represented His6MBP-tevS-eDAL, His6MBP-tevS-sDAL, His6GST-tevS-eDAL, His6GST-tevS-sDAL, His6-tevS-eDAL and His6-tevS-sDAL. **B**. Purification of the fusion proteins. The fusion proteins were purified by Ni-NTA and analyzed by SDS-PAGE. All the proteins are indicated by arrows. **C**. The activity of the DAL as the fusion partner in the crude extracts. **D**. The activity of the purified fusion proteins for DAL.

To first investigate the initial solubility of the DAL-fusion protein, we created a T7 promoter expression vectors for the production of the fusion proteins containing sequences encoding His6MBP, or His6GST or His6 tags followed by a TevS (TEVp cleavage sequence) and multiple cloning sites, respectively. Sequences encoding eDAL or sDAL were then cloned into the MCS to create an in-frame fusion of DAL with each tag. All the constructed proteins were expressed at high level in *E. coli*, as detected by Western blot analysis using anti-His6 antibody (Figure 1A). His6MBP tag was the most efficient on improving the protein solubility of DAL. The DAL-fusion proteins were purified by Ni-NTA affinity chromatography and displayed relatively high purity on SDS-PAGE analysis (Figure 1B), suggesting that the fusion proteins were stable in the solution.

We then measured activity of the DAL-fusion proteins from the crude extract and the purified form (Figure 1C and 1D). These three sDAL-fusion proteins showed different activities in the crude extract. Similar difference was also found in the purified forms. This was phenotypically consistent with the expression level of sDAL in *E. coli* (Figure 1A). Interestingly, difference in activities of eDAL-fusions from crude extract was distinct from that of their purified forms, probably due to the various expression levels.

### Effects of protease cleavage sequence on production and activity of the fused eDAL

**Figure 2:**
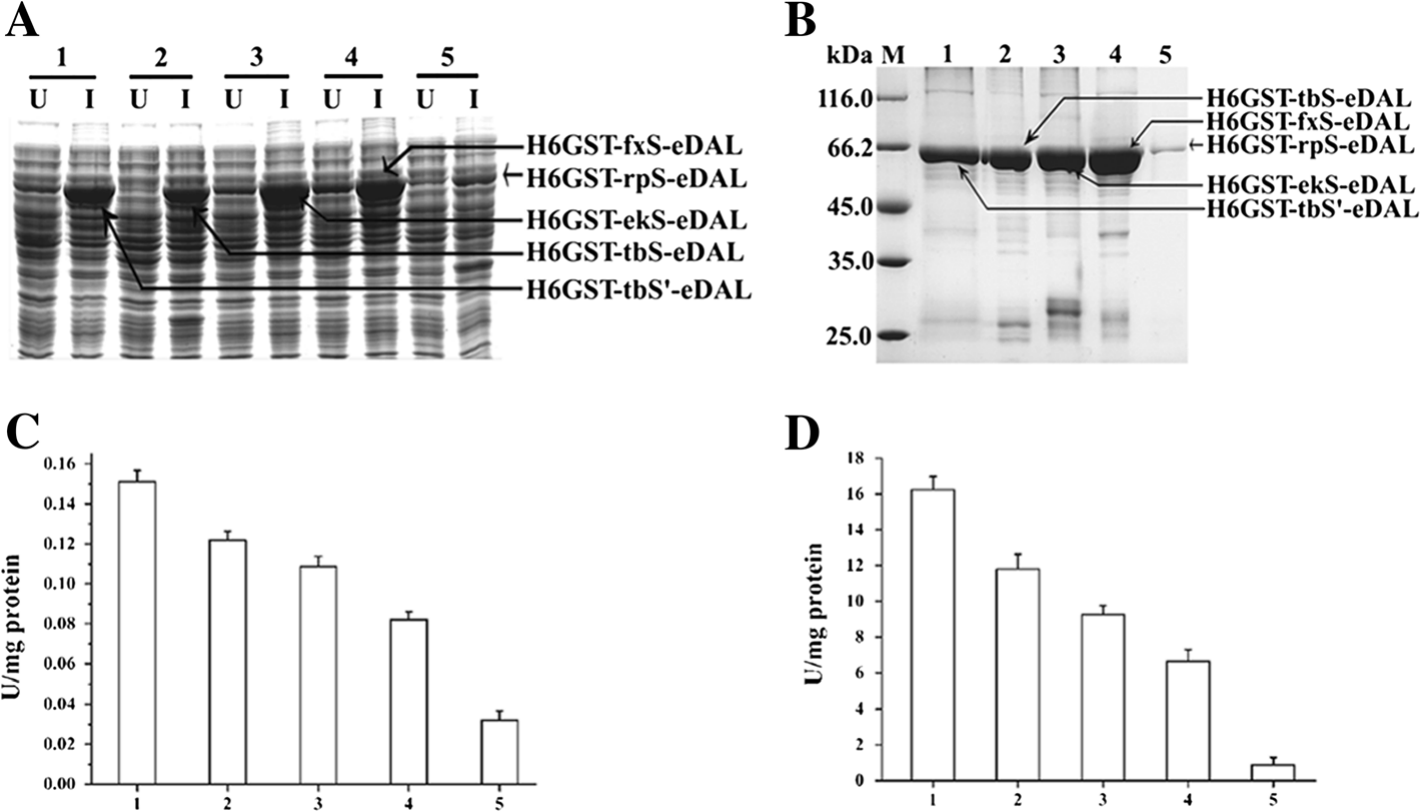
**Overexpression**, **purification and activity assay of eDAL with a His6GST followed by the specified protease cleavage site. A**. SDS-PAGE analysis of the soluble fusion proteins expressed in *E. coli*. U: uninduced; I: induced. **B**. SDS-PAGE analysis of the purified fusion proteins. In the Figure 2**A** and 2**B**, all the proteins are indicated by arrows. **C**. DAL activity of the fusion protein in the crude extracts. **D**. DAL activity of the purified fusion protein.

To determine the effect of protease cleavage sequence on the DAL-fusion protein, 5 different protease recognition sequences, including enterokinase sequence DDDDK (ekS), factor Xa sequence IEGR (fxS), R3P sequence LEVLFQG (rpS) etc., were used to link His6-GST tag and eDAL. For thrombin, two sequences containing either glycine (Gly) or serine (Ser) at the P1’ position of the thrombin recognition sequence (LVPRG/S, termed tbS or tbS’) were also used. Expression of designed DAL-fusion proteins was determined by SDS-PAGE (Figure 2A). All the fused eDAL proteins were purified and analyzed by SDS-PAGE analysis (Figure 2B). All fusion proteins had high expression in *E. coli*, except the His6GST-rpS-eDAL. Activity test using the crude extract showed that these fusion proteins again had different DAL activity (Figure 2C), which are consistent with the difference observed for their purified forms (Figure 2D). Incorporation of R3P cleavage sequence (rpS) severely decreased protein expression level and DAL activity.

### Resistance of DAL Activity to protease activator and inhibitor

Proteases are classified according to the presence of catalytic residues including Ser, Cys, Thr, or Glu. The proteases wildly used for tag removal belong to serine and cystein proteases. Phenylmethanesulfonyl fluoride (PMSF) and benzamidine hydrochloride (BzCl) are specific inhibitor of serine protease. N-ethylmaleimide (NEM) is an inhibitor specific for cysteine protease. On the contrary, Ca^2+^ can activate several proteases including factor Xa and thrombin [3]. Since different proteases demand different buffer compositions for sustainable solubility, stability, and biological activity, it is extremely important to pre-determine whether DAL activity is resistant to different conditions, particularly to these protease activators and inhibitors. Therefore, we measured the DAL activity using the characterized eDAL-fusion proteins in the presence of calcium ion, EDTA, and specific protease inhibitors including NEM, PMSF and BzCl. All reagents at tested concentrations slightly activated eDAL, but the differences were within 10% (Table 1). Thus, the results suggest that using eDAL-fusion to measure the protease activity under various conditions is practicable.

**Table 1:** The effect of some reagents on DAL activity

**Reagent**	**Relative activity %**
**CK**	**100.00**
**5 mM EDTA**	**107.79**
**2.5 mM Ca**^**2+**^	**104.43**
**1 mM NEM**	**101.67**
**1 mM BzCI**	**109.20**
**1 mM PMSF**	**109.74**

### Application of eDAL-fusion coupled activity assay for sequence-specific proteases

**Figure 3:**
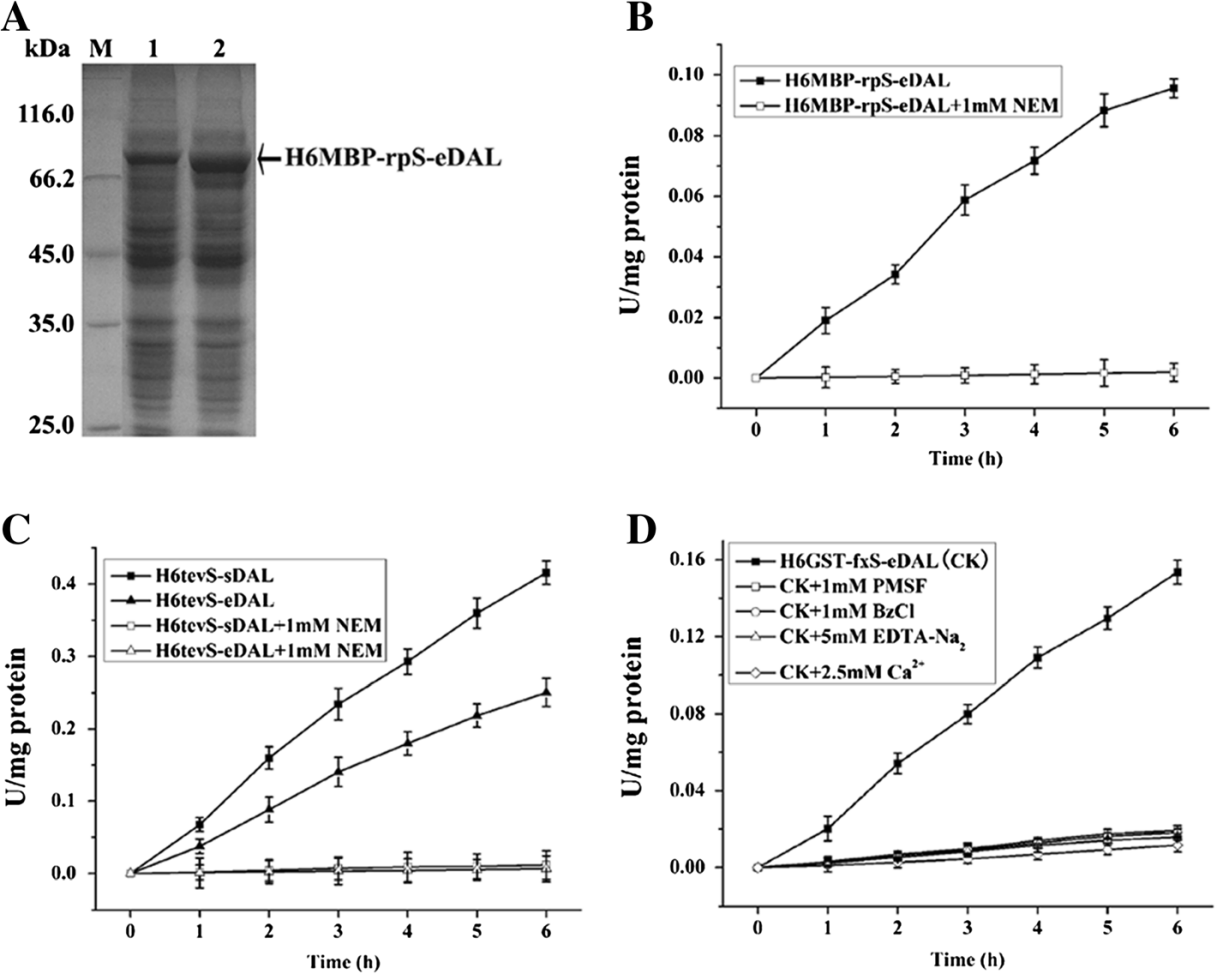
**Coupled assay of time course cleavage of the designed fusion protein by proteases. A**. SDS-PAGE analysis of expressed His6MBP-rpS-eDAL in *E. coli* cells. U: uninduced; I: induced. The protein is indicated by the arrow. **B**. Time course cleavage activity of His6MBP-rpS-eDAL from crude extracts using GST fused R3P by coupled assay in the absence or presence of the inhibitor. **C**. Coupled assay of time course cleavage of two fusion proteins for eDAL and sDAL from crude extracts by the TEVp variant. **D**. Coupled assay of time course cleavage of the fusion protein from crude extracts by factor Xa. The activity of DAL partner was used as a control and subtracted.

We further determined whether eDAL-fusion proteins can be used to monitor the protease activity through their enhanced DAL activity after cleavage. First, we optimized the His6GST-rpS-eDAL that was found to be poorly expressed in *E. coli* in our previous test. We substituted His6GST with His6MBP in the fusion protein. SDS-PAGE analysis showed it with enhanced expression level in the cells (Figure 3A). This His6MBP-rpS-eDAL fusion protein from the crude extract was also cleaved efficiently by R3P incubated for different times. In addition, adding the thiol alkylating agent NEM that inhibits cysteine proteases (such as R3P and TEVp) abolished the function of R3P and the cleavage of the eDAL-fusion protein (Figure 3B), supporting the specificity of optimized His6MPB-rpS-eDAL fusion substrate.

Enzyme assay monitoring the activity of DAL also support the efficient proteolysis of His6-tevS-eDAL and His6-tevS-sDAL by a TEVp variant. DAL activities increased with reaction time, and His6-tevS-sDAL had more sensitivity than His6-tevS-eDAL. Again, in the presence of NEM, the TEVp activity was inhibited and no increase of DAL activity was observed when incubated with TEVp (Figure 3C).

Factor Xa cleaved the corresponding His6GST-fxS-eDAL at a relatively high speed under standard condition. Factor Xa is a serine protease that can be inactivated by PMSF and BzCl. In addition, calcium ions bind to the enzyme and function as cofactor for proteolysis [3]. Again DAL activities were much decreased in the presence of PMSF, BzCl or EDTA (Figure 3D).

### Cleavage specificity of DAL-fusion proteins as thrombin and enterokinase substrates

**Figure 4:**
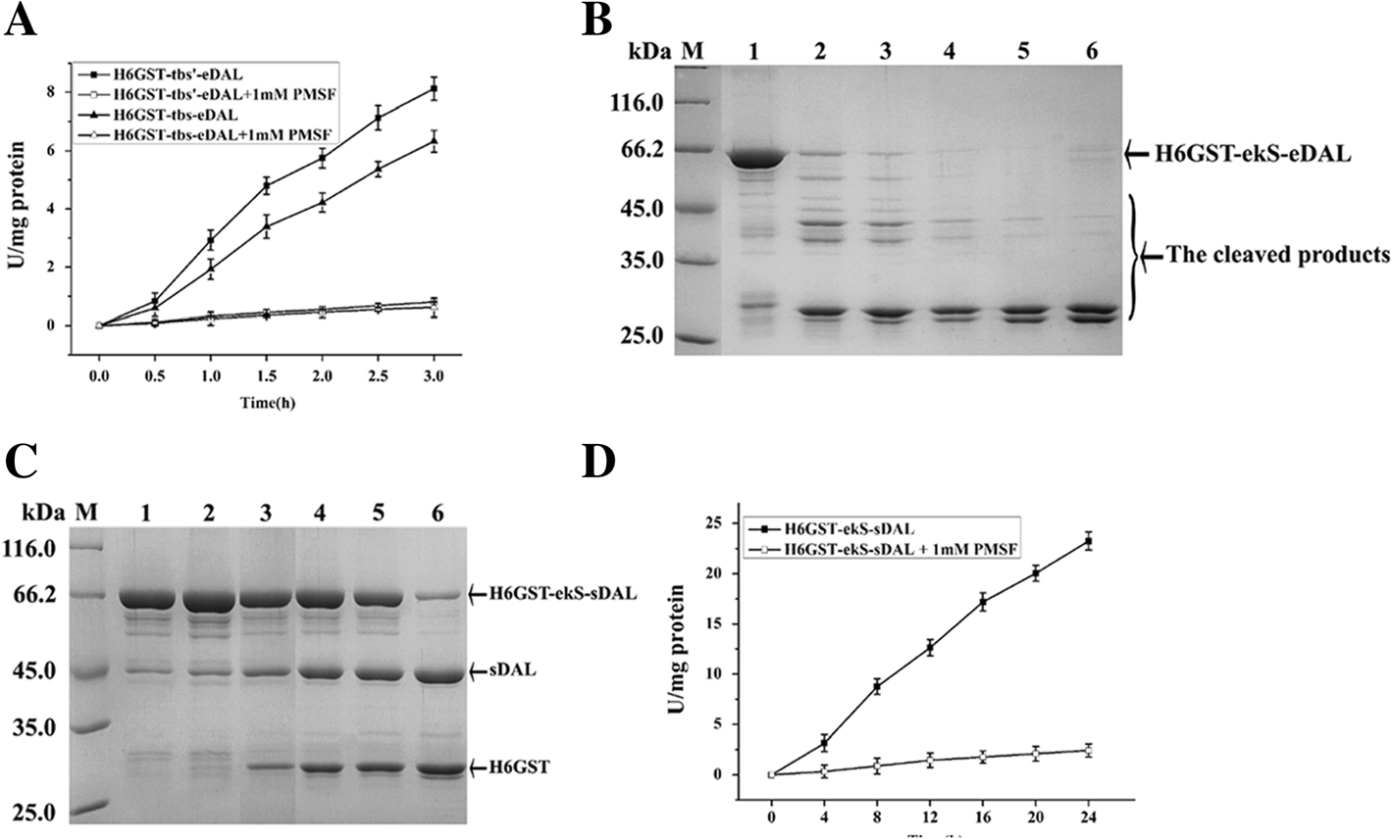
**Substrate preference and specificity of thrombin and enterokinase by the coupled assay. A**. coupled assay of fusion protein containing tbS or tbS’ cleaved by thrombin in the absence or presence of PMSF. **B**. SDS-PAGE analysis of time course cleavage of the purified fusion protein for eDAL at undesired cryptic sites by enterokinase. The fusion protein is indicated by the arrow. **C**. The purified fusion protein for sDAL was cleaved into two parts by enterokinase. The fusion protein and cleaved products are indicated by the arrows. **D**. coupled assay of time course cleavage of the purified fusion protein for sDAL by enterokinase. The activity of DAL partner was used as the control and subtracted.

To further determine if the DAL-fusion proteins can also be used to test the specificity of proteases, two thrombin cleavage sequences (tbS and tbS’) were used as linker between His6-GST and eDAL. Proteolysis-DAL activity coupled assay showed that thrombin had about 1.3 fold more processing efficiency for LVPRS than LVPRG (Figure 4A), with strong preference of S at the P’1 position, as previously shown using synthetic fluorescence peptide probes [24]. Further, adding PMSF abolished the increase of DAL activity presumably by inhibiting thrombin for both DAL-fusion substrates (Figure 4A). This result supports the idea that our designed DAL-fusion protein can display similar substrate specificity as classical synthetic substrate probe for protease [24].

Another interesting result was seen for His6GST-ekS-eDAL fusion as substrate of enterokinase. Unexpectedly, after cleavage of the fusion protein, the activity of the released eDAL was very low (data not shown). SDS-PAGE analysis denoted that eDAL was cleaved by enterokinase (Figure 4B). When eDAL was substituted with sDAL, the fusion protein was only cleaved in two parts, His6-GST and sDAL (Figure 4C). Consistently, proteolysis-DAL coupled assay showed that, with prolonged time of enterokinase incubation, sDAL activity increased. Adding PMSF inactivated the DAL activity presumably by inhibiting the enterokinase-directed proteolysis (Figure 4D). The selected DAL from the special organism is suitable for analysis of the activity of enterokinase.

### Application of DAL-fusion substrate for intein self-cleavage activity

**Figure 5:**
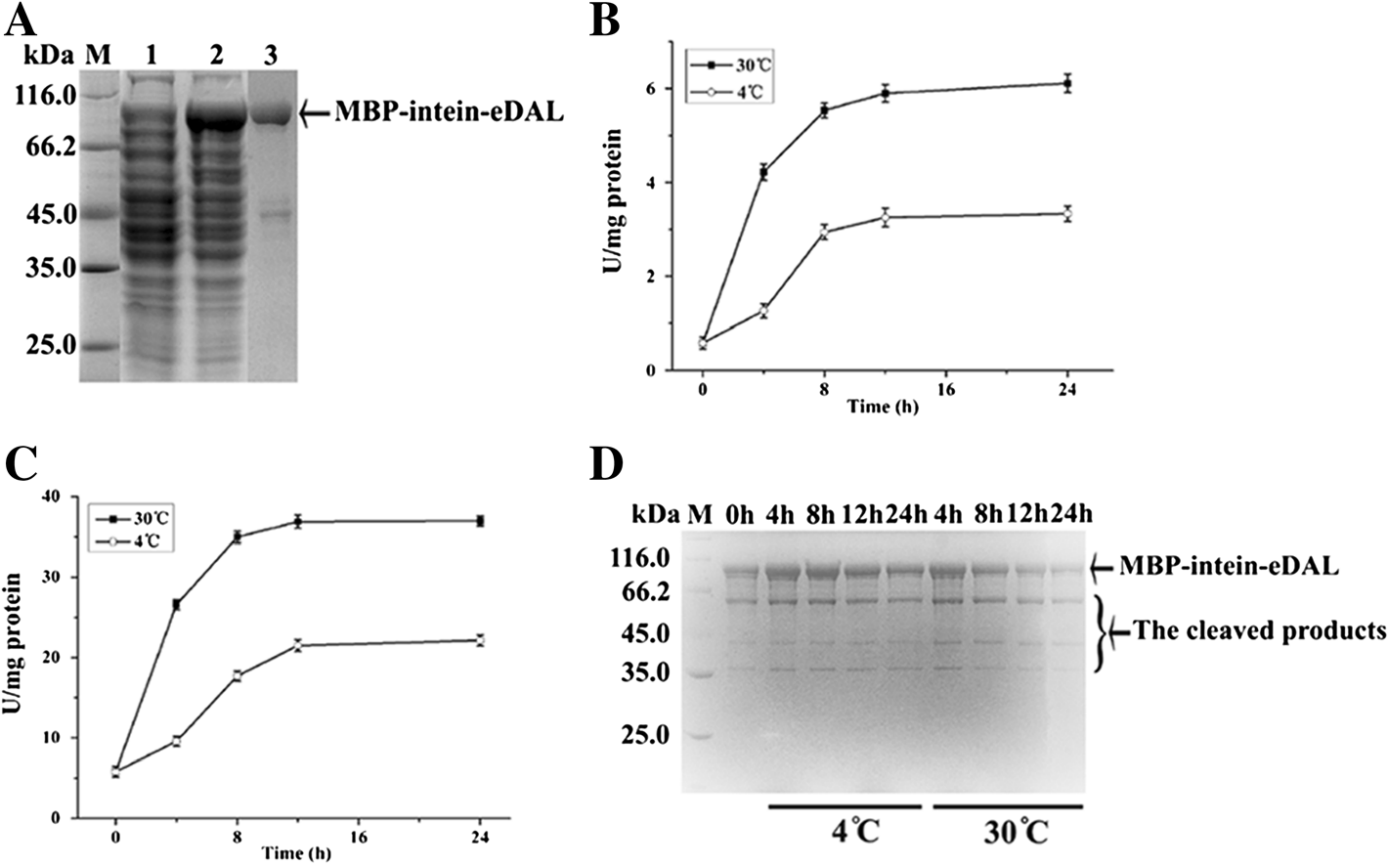
**Self**-**cleavage ef**fi**ciency of MBP**-**intein**-**eDAL at pH 6.5 and at 4°C and 30°C respectively. A**. SDS-PAGE analysis of expressed and purified fusion protein. The specific bands were indicated by the arrow. **B** and **C**, self-cleavage activity of fusion protein at different incubation time from crude extract and in purified form were analyzed by coupled assay. **D**: self-cleavage of fusion protein at corresponding incubation time detected by SDS-PAGE analysis.

We tested the possibility of using DAL-fusion protein as substrate for the self-cleavage reaction. To increase the fusion protein expression level, we constructed a chimeric protein using His6-MBP, Ssp DnaB intein and eDAL. This MBP-intein-eDAL had much improved expression of the soluble protein in *E. coli*, and was purified with high purity (Figure 5A). The fusion protein from the crude extract and in the purified form was cleaved at pH 6.5, 4°C and 30°C. The released DAL activity was measured at pH 8.0 at 37°C for 5 min to access the self-cleavage efficiency (Figure 5B and 5C). DAL activity increased dramatically between 0 and 12 h, dropped slightly between 12–24 h, which corresponded with the self-cleavage of the purified fusion protein as shown by SDS-PAGE analysis (Figure 5D). The result also indicates that the intein-DAL coupled activity assay could measure the intein self-cleavage activity in cell lysate and does not require purified protein.

### Application of DAL-fusion substrate for SUMO protease in vitro and in E. coli cells

**Figure 6:**
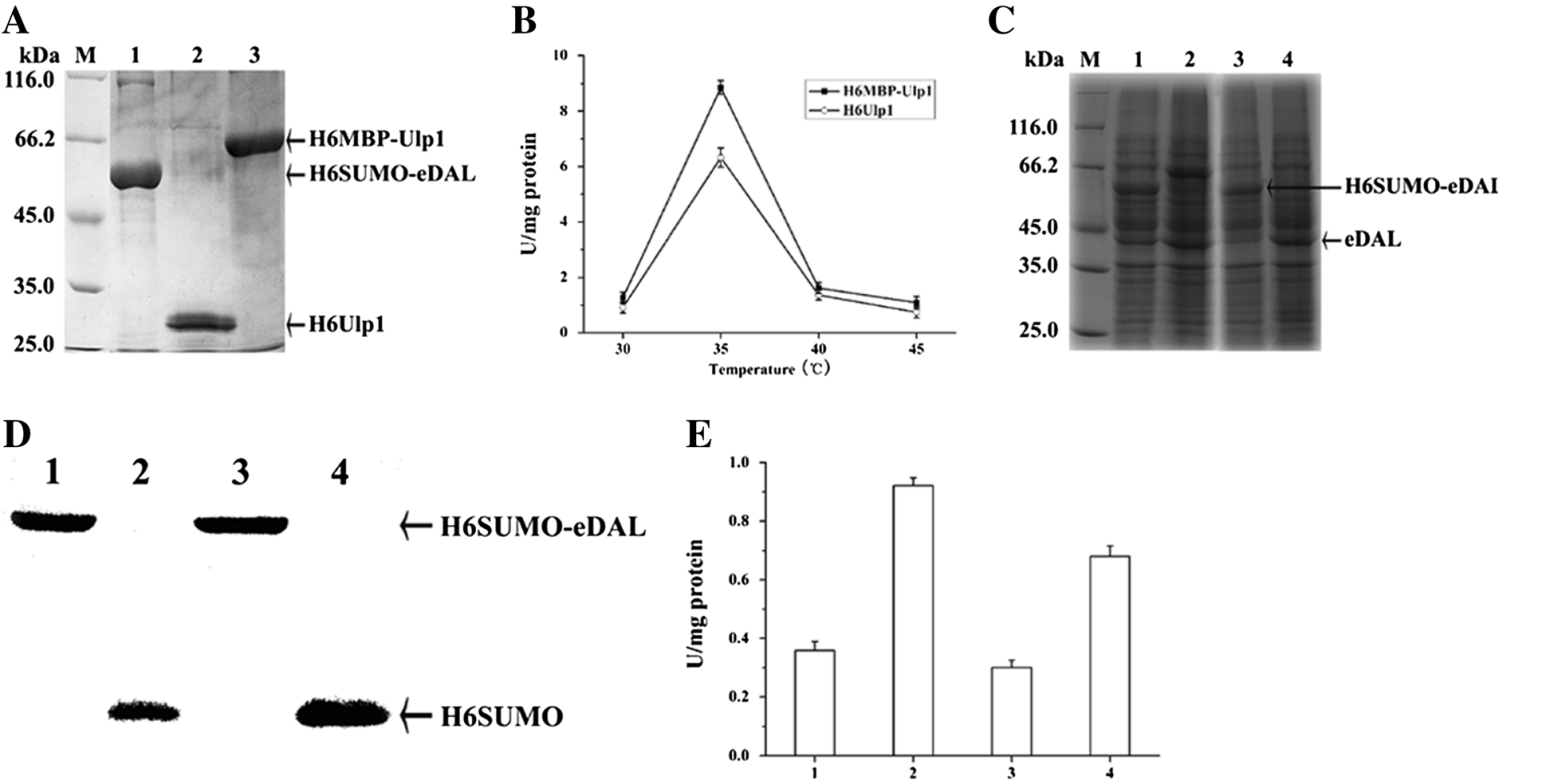
**In vitro and in vivo cleavage of His6SUMO**-**eDAL by the Ulp1 constructs. A** SDS-PAGE analysis of the purified fusion protein and two Ulp1 constructs (His6MBP-Ulp1 and His6-Ulp1). The corresponding proteins are indicated by the arrows. **B**: in vitro cleavage of SUMO-eDAL by two Ulp1 constructs at the different temperatures by coupled assay. **C**-**E**: in vivo cleavage of SUMO-eDAL by Ulp1 constructs detected by SDS-PAGE and Western blot analysis, and activity measurement of the released DAL. Lane1: cells carrying the plasmids pSUMO-eDAL and pMAL-c2x. Lane 2: cells containing pSUMO-eDAL and pMAL-c2x-Ulp1. Lane 3: cells harboring pSUMO-eDAL and pCDF-Duet1. Lane 4: cells carrying pSUMO-eDAL and pCDF-Ulp1. The expressed His6SUMO-eDAL and cleaved His6SUMO were indicated by the arrows. The colume in Figure 6E represented the corresponding DAL activities. The in vivo and in vitro activity of DAL as the fusion partner was applied as the control and subtracted.

We also tested the possibility of using DAL-fusion protein as substrate for SUMO protease Ulp. Two yeast Ulp1 constructs were investigated. First, the constructed protease and protein substrate were purified by Ni-NTA to homogeneity (Figure 6A). Different from previous observation [5], the purified His6-tagged Ulp1 with a His6-tag became completely precipitated and inactivated in buffer (50 mM Tris/HCl, 100 mM NaCl, pH 8.0) after storing at -20°C overnight, but the HisMBP-tagged Ulp1 was stable and used for most of the analysis. Using the fusion protein His6SUMO-eDAL as a substrate, we showed that Ulp1 activity was temperature dependent. Ulp1 activity was increased from 30 to 35°C, but became decreased after 35°C, with the maximum activity at 35°C. For fresh protease, His6MBP tagged Ulp1 was more active than His6 tagged Ulp1 at 35°C, while the activities were almost equal at temperature between 35–45°C (Figure 6B).

Finally, we would like to check whether Ulp1 activity could be quantitatively measured in vivo using the fusion protein substrate. We co-expressed His6- or His6MBP tagged Ulp1 together with the protein substrate in *E. coli*. The cleavage of the fusion protein was monitored by SDS-PAGE and the DAL activity assay. Ulp1 could cleave the protein substrate in *E. coli* cells, as shown by SDS-PAGE and Western blot analysis (Figure 6C and 6D). Consistently, higher DAL activities were observed in these cells than the control cells that harbored pSUMO-eDAL vector and the control plasmid (the plasmid without insertion of the gene encoding Ulp1). Furthermore, cells expressing His6MBP-Ulp1 showed higher DAL activity than cells expressing His6-Ulp1 (Figure 6E). These observations support that the activity of Ulp1 could be measured by cleaving the protein substrate through the activity of liberated eDAL in *E. coli*.

## Discussion

Fusion protein substrates are important tools for quantitatively measuring the activity of proteases, and can be used for protease engineering to improve specificity, solubility and folding, and for quick protease activity assay. Several fusion protein substrates have been developed before, but a more sensitive and inexpensive substrate that can be used both in vitro and in cells is still lacking. Here, based on the finding that N-terminal tagged eDAL and sDAL had much lower DAL activity (3.9 and 4.0 U/mg) than the untagged enzymes (80 ± 20 and 190 ± 20 U/mg) [22], we propose that the fused DAL might be suitable as protease substrate and the proteolysis-DAL coupled assay could be used to quantitatively determine the protease activity. We show, unlike some other protein substrate produced in *E. coli*, DAL can be fused with different tags. A select group of tags provided sufficient yield and inhibition of eDAL and sDAL activity. Moreover, we also optimized several fusion tags that had further improved yield and activity of DAL proteins to allow directly measuring the DAL activity even in cell lysate. Without purification, sDAL-fusion proteins provided higher sensitivity than that of eDAL-fusion in this proteolysis-DAL coupled assay. Thus, our results indicate that the recombinant DAL-fusion proteins can be developed as a common protein substrate platform for directly analyzing protease activity.

Fusion tags acted differently on the production and stability of the target proteins with the incorporated cleavage sequence [25]. Several approaches can be applied to overcome this problem. For example, in this study, we found that inclusion of an rpS greatly decreased the expression of fusion protein. Attempts to enhance the expression level of His6GST-rpS-eDAL at various growth temperatures and induction by IPTG at different concentration were failed. However, substitution of His6-GST with His6-MBP considerably improved the expression of the fusion protein (Figure 3A). The low expression of His6GST-rpS-eDAL may not be caused by some universal feature of rpS. In fact, the pGEX vectors are commercial available for expression of GST-fusion protein containing rpS under control of a tac promoter. Our observation suggests that rpS may have specific effect with the DAL and GST sequence. Fusion protein substrates for different protease should be optimized specifically even when a universal protein platform is used, such as DAL.

In addition to the gain of activity after proteolysis, DAL shows other important features as ideal protease substrate. First, DAL activity is insensitive to all tested protease specific inhibitors such as PMSF, BzCl and NEM. These inhibitors modify the serine and cysteine residues to inactive proteases either covalently or noncovalently, while two residues (Lys77 and Asp120) are potential catalytic residues as determined in the eDAL structure [26]. Second, DAL activity also remains constant with the presence of metal ions such as Ca^2+^ or metal ion chelators such as EDTA, which are required for protease activity. Thus, the orthologous reaction of DAL poses it as a great candidate to monitor protease activity in various experimental conditions.

DAL-fusion also provides a platform to identify specificity of the target protease. Serine proteases such as thrombin, enterokinase and factor Xa are widely applied for removing the fusion tag. Accumulated evidence indicates that these proteases showed relatively low stringent specificity and often cleave the fusion protein at locations other than the designed site and development of next generation of serine proteases with higher substrate is desired [4].Thrombin has a preference for serine at P1’ position [4]. Consistently, our DAL-fusion substrates showed that the specific activity of thrombin for cleaving LTPRS is nearly one and half folds of that for LTPRG. Further, enterokinase exhibits a preference for substrates with motif D/ERM, as well as LKGDR, which are cleaved more efficiently than the canonical DDDDK recognition sequence [27,28]. In this study, we found that enterokinase cleaved both eDAL and sDAL substrates but yielded no DAL activity in eDAL. In-depth analysis showed that enterokinase also cleaved the eDAL at some cryptic sites. The possible enterokinase cleavage sequence was deduced in eDAL, and only VKGD amino acids similar to the reported LKGDR are different from the alignment of sDAL and eDAL sequences (See Additional file 2: Figure S2). Based on our research, the designed DAL-fusion protein substrate could be applied to analyze the known protease specificity, but also be useful in detecting unidentified substrate specificity of target proteases.

Application of DAL-fusion platform is not limited to simple proteolysis. Self-cleaving tags in fusion proteins possess inducible proteolytic activities. They enable fusion purification, cleavage and target protein separation to be achieved in a single step and are widely used in protein production [6]. Induction of the intein-mediated cleavage can be achieved by thiol reagents or pH and/or temperature shift, depending on the particular construct used [6]. This has allowed many proteins (as N-terminal fusion) to be overexpressed and purified using Ssp DnaB intein as self-cleavage tag. A direct approach to monitor the efficiency of self-cleavage has not been developed. We showed the DAL-fusion platform could be applied for this purpose. Further, we showed a His6-MBP tag could dramatically increase expression level of the fusion through direct examination of the intein activity by the proteolysis-DAL coupled assay. This finding is of particular application for other target proteins (instead of eDAL), which His6-MBP tag will allow it to be directly purified by cleaving the chimeric protein on either Ni-NTA or amylase resin. In contrast, while purification tags including chitin binding domain, Phasin, phaR, ELP, His6 and cellulose-binding module had been used together with Ssp DnaB intein, they do not often lead to soluble proteins in *E. coli*[6].

SUMO-specific proteases represent a special class of protease that recognizes a much larger substrate identity. SUMO-specific proteases such as Ulp1 recognize the C-terminal end of SUMO and hydrolyze peptide or isopeptide bonds to the carboxyl group of the C-terminal glycine residue [5]. Here we show that DAL-fusion platform can also be used as substrate to monitor SUMO protease activity. The cleavage of SUMO-eDAL by two Ulp1 constructs showed different DAL activities, supporting the difference of these two Ulp1 enzymes. Because of the shared mechanism between SUMO and ubiquitin transference, in the future, by exchanging SUMO with ubiquitin, it might be possible to analyze the conformational-specific de-ubiquitin enzymes using DAL-fusion platform [5].

Up to now, a few specific proteases have been produced in *E. coli*, while further engineering of these protease are needed to overcome their impaired folding and less solubility. Several strategies have been exploited to improve the intrinsic defects [4], for example, the change of some amino acid residues on the surface of TEVp and human enterokinase by site-directed mutagenesis [29,30], which all require to quantitative measure the protease activity. Our developed DAL-fusion protease substrate will provide a rapid and simple approach of assaying sequence- and conformational-specific protease activity.

## Materials and methods

### Materials

Amylase resin, the plasmids pMAL-c2x and pTWIN1 are products of NEB, USA. Ni-NTA and proteases including factor Xa, enterokinase and thrombin are bought from Novagen, USA. *E. coli* strain BL21(DE3), and pET28b and pCDF-Duet1 vectors, and reagents for Western blot analysis are supplied by Novagen, USA. Ni-NTA superflow was obtained from Qiagen (Chatsworth, CA). Ultra-15 centrifugal filter tube equipped with Ultracel-10 membrane was supplied by Amicon (USA). The compounds including 2,4-dinitrophenylhydrazine (2,4-DNP), PLP and DL-DAP, PMSF, NEM, BzCl were from Sigma. Primer synthesis and DNA sequencing were performed by Invitrogen (Shanghai, China). Reagents used in plasmid construction and protein expression were from Takara (Dalian, China). The purified GST fused R3P is generously provided by Professor Zang Jianye, University of Science and Technology of China. The TEVp variant with five amino acid mutations are purified previously [23].

### Plasmids construction

The plasmid pGST-eDAL for expression of eDAL with a His6GST followed by a TEVp cleavage sequence was constructed [23]. The gene encoding sDAL was amplified by PCR using *S. typhimurium* genomic DNA as the template and substituted the sequence encoding eDAL to yield pGST-sDAL. Further, His6GST was replaced with either His6MBP or His6-tag. By overlap PCR, the recognition sequence for TEVp was exchanged by that for other protease including ekS, fxS, rpS, tbS and tbS’. The plasmid pMID encoding the MBP-intein-eDAL fusion protein was constructed. The gene fragment encoding Ssp DnaB intein was amplified by PCR using pTWIN1 vector as the template, and inserted into pMAL-c2x. Then, the gene encoding eDAL was subcloned into downstream of coding sequence of Ssp DnaB intein. The eDAL gene was also amplified, digested with the specified restrictive endonuclease, and inserted into pTYB21 vector. The *sumt3* and *Ulp1* were amplified using *Saccharomyces cerevisiae* genomic DNA as the template, based on the published literature [2]. The amplicon was cut and inserted into pET28b, to generate the plasmids p28SUMO and p28Ulp1 respectively. The gene encoding eDAL was cut from pGST-eDAL and inserted into p28SUMO to create the plasmid pSUMO-eDAL. The *Ulp1* in the plasmid p28Ulp1 was excised and subcloned into pCDF Duet I to yield pCDF-Ulp1. This gene was also amplified and inserted into pMAL-c2x, to generate the plasmid pMAL-Ulp1 for expressing MBP-Ulp1 fusion protein. The primers were listed in Additional file 3: Table S1. All the amplified gene fragments were sequenced.

### Overexpression and purification of the fusion proteins

The constructed plasmid was transformed into BL21(DE3). The transformants were cultured overnight at 37°C and diluted 1:200 in LB culture. When OD_600_ were up to 0.5 at 37°C, IPTG at final concentration of 0.5 mM was added and the cells were grown overnight at 28°C. Cells were collected by centrifugation and dissolved in buffer A buffer A (20 mM Tris/HCl, 100 mM NaCl, pH 8.0), and disrupted by sonication at 4°C. After centrifugation, the supernatant was collected. Protein concentration was determined by coomassie brilliant blue R250, using bovine serum albumin as standard. Soluble fractions were separated on 15% SDS-PAGE gel and electro blotted onto PVDF membrane. The membrane was blocked with PBST (10 mM phosphate buffer with 150 mM NaCl, 0.05% Tween 20 and 5% skimmed milk) for 40 min at room temperature, and then incubated for 2 h with anti-His6 or SUMO mouse monoclonal antibody diluted in PBST with 5% skimmed milk. After three washes with PBST buffer, the membrane was incubated for 1 h with horseradish peroxidase conjugated anti-mouse IgG diluted in PBST with 5% skimmed milk, washed three times and specific proteins were visualized by adding 4-chloro-1-naphthol solution dissolved in 20% methanol and 0.08% hydrogen peroxide in PBST.

The cells overexpressing the fusion protein were also disrupted in buffer B (50 mM sodium phosphate, 300 mM NaCl and 10 mM imidazole, pH 8.0). The supernatants were loaded on a 3-ml Ni-NTA resin packed in a 15 ml column, pre-equilibrated with three column volumes of buffer B, then washed and eluted with three column volumes of 30 and 250 mM imidazole in buffer B. The MBP-Ulp1 was purified by amylase resin according to NEB protocol. All the purified fusion proteins were concentrated, exchanged with buffer A, and analyzed by 15% SDS-PAGE.

### In vitro and in vivo cleavage of the designed fusion protein

The Ulp1, TEVp, or rhinovirus 3C protease in buffer A, thrombin in buffer C (20 mM Tris–HCl, 100 mM NaCl, and 1 mM CaCl_2_, pH 7.5), factor Xa or enteropeptidase in buffer D (20 mM Tris–HCl, 100 mM NaCl, and 1 mM CaCl_2_, pH 8.0), cleaved the corresponding fusion protein at 25°C. About 0.5 ml of crude extracts of *E .coli* cells overexpressing the fusion protein were mixed with the different concentration of protease solutions. At the indicated time points, aliquots were withdrawn and the activity released DAL was analyzed. The activity of the fusion protein for DAL was assayed and subtracted. The corresponding samples were boiled for 5 min and analyzed by SDS-PAGE.

*E. coli* BL21 (DE3) overexpressing SUMO-eDAL and Ulp1 with a N-terminal His6-tag or MBP were cultured overnight at 37°C in the presence of the corresponding antibiotics. Cells harboring the plasmids pSUMO-eDAL and pCDF-Ulp1 or pMAL-Ulp1 (The plasmid pCDF-Duet1 or pMAL-c2x as control) were collected, disrupted in buffer A, and centrifuged. The supernatants were analyzed by SDS-PAGE. The DAL activity was assayed.

### DAL activity analysis

DAL activity was measured in the absence or presence of each of the reagents including 2.5 mM Ca^2+^, 5 mM EDTA-Na_2_, 1 mM NEM, 1 mM PMSF and 1 mM BzCl. DAL catalyzes DL-DAP to generate pyruvate and ammonia. The amount of pyruvate was measured with 2,4-DNP. The reaction mixture in 1 ml contained 50 μM PLP and 10 mM DL-DAP and the protein. The assay was started at 37°C for 5 min and stopped by adding 1 ml of 2 mM HCl containing 0.03% 2,4-DNP. The mixture was incubated at 4°C for 5 min, and 2 ml of 2 M NaOH was added. Absorbance at 520 nm was recorded on a U-2001 spectrometer (Hitachi, Japan). The experiments were carried out in triplicates. One unit of enzyme activity was defined as the amount of enzyme required to catalyze the release of 1 μmol of pyruvate/min at 37°C, pH 8.0.

## Competing interests

The authors declared that they have no competing interests.

## Authors’ contributions

CZ constructed the expression plasmids for expressing several fusion proteins and analyzed activity of all proteases. YY constructed the plasmids for expressing the fusion proteins for sDAL. JFang constructed the plasmid for expressing GST fused eDAL and purified the recombinant TEV protease. BC supplied the platform for the experiment. JFan designed the experiment and wrote the paper. All the authors approved the final manuscript.

## Supplementary Material

Additional file 1: Figure S1 The expression level of Sce-VMA1-CBD-intein-eDAL by SDS-PAGE analysis. The fusion protein was overexpressed in *E. coli* BL21(DE3) under induction with 0.5 mM IPTG for 12 h at 28°C, and indicated by the arrow. Click here for file

Additional file 2: Figure S2 Amino acid sequence alignment of eDAL and sDAL. The deduced cleavage site for enterokinase in the loop of eDAL was labeled in red color. Figure was prepared with programme CLUSTAL W. Click here for file

Additional file 3: Table S1 The primers used in this study. Click here for file
