# Supplementary figures and images for: A new fusion protein platform for quantitatively measuring activity of multiple proteases
Source: Microb Cell Fact. 2014 Mar 21;13:44. doi: 10.1186/1475-2859-13-44 (PMC4000059; doi:10.1186/1475-2859-13-44)

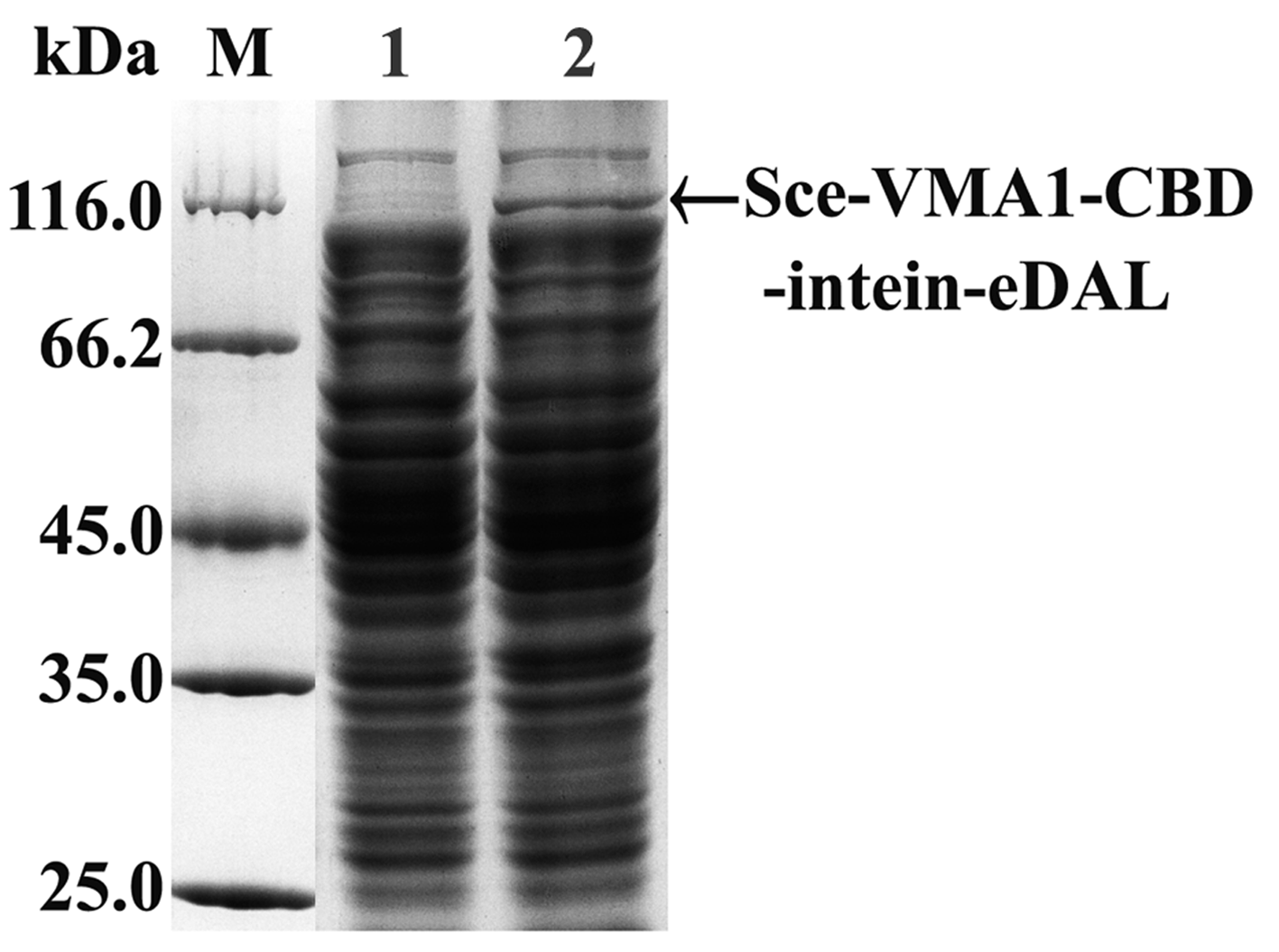

Supplement: Additional file 1: Figure S1 — The expression level of Sce-VMA1-CBD-intein-eDAL by SDS-PAGE analysis. The fusion protein was overexpressed in E. coli BL21(DE3) under induction with 0.5 mM IPTG for 12 h at 28°C, and indicated by the arrow. [file 1475-2859-13-44-S1.tiff]

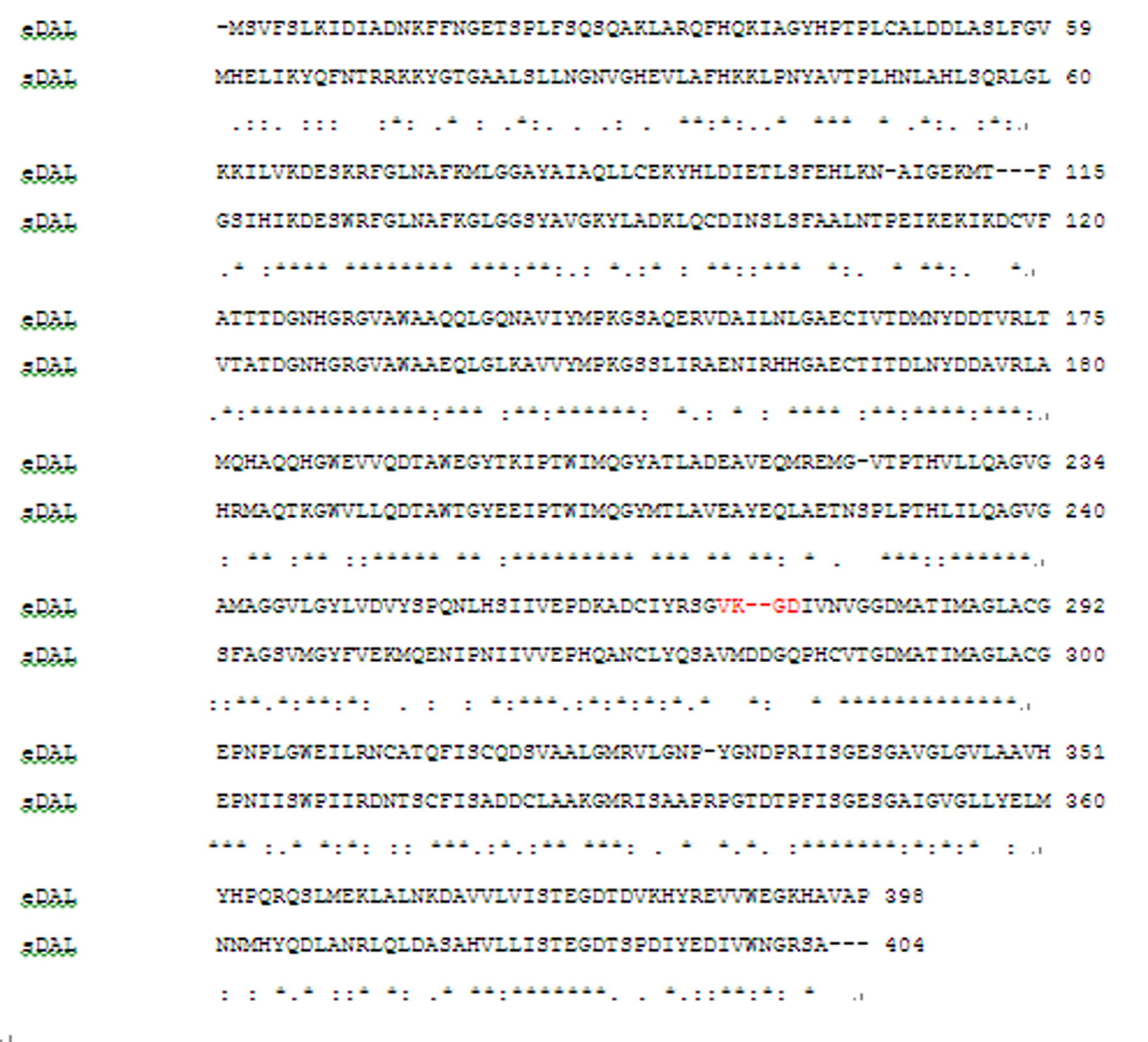

Supplement: Additional file 2: Figure S2 — Amino acid sequence alignment of eDAL and sDAL. The deduced cleavage site for enterokinase in the loop of eDAL was labeled in red color. Figure was prepared with programme CLUSTAL W. [file 1475-2859-13-44-S2.tiff]
