# Supplementary material for: A new fusion protein platform for quantitatively measuring activity of multiple proteases
Source: Microb Cell Fact. 2014 Mar 21;13:44. doi: 10.1186/1475-2859-13-44 (PMC4000059; doi:10.1186/1475-2859-13-44)
Supplement: Additional file 3: Table S1 — The primers used in this study. [file 1475-2859-13-44-S3.doc]

**Table S1** Primers used in this study

| Primer | Sequence (5’→3’) | Usage |
| --- | --- | --- |
| ekS1  ekS2  fxS1  fxS2  rpS1  rpS2  eDAL2  tbS’1  tbS’2  ssp1  ssp2  sDAL1  sDAL2  sumo1  sumo2  Ulp1  Ulp2 | CTGAGATCTGACGACGACGACAAGGGATCC  GACGACGACAAGGGATCCGTTTTCTCATTGAAG  CTGAGATCTATCGAAGGTCGTGGATCC  ATCGAAGGTCGTGGATCCGTTTTCTCATTGAAG  CTGAGATCTCTGGAAGTTCTGTTCCAAGGTCCAGG  GTTCCAAGGTCCAGGATCCGTTTTCTCATTGAAG  TGTAAGCTTAAGGTGCTACAGCGTGTTTG  TCTCGGAACCAGACTAGCTTTTGGAG  GGATCCGTTTTCTCATTGAAG  TCGAGCTCGAACGGTAACAACGGTCTCGAACTG  CGTGGATCCGTTGTGTACAATGATGTCATTC  TCGGATCCCATATGCATGAGCTTATTAAATATC  GATCTCGAGTTAAGCACTGCGTCCGTTCCAGACT  TATGTCATATGCACGGGTCGGACTCAGAAGTC  TAATGGATCCGGTCTCAACCTCCAATCTGTTC  ATGGGATCCCATATGCAGATCTTTGTGAAGACC  GATAAGCTTAACCTCCTCGCAGGCGCAACACCAG | Overlap PCR    Overlap PCR    Overlap PCR  Amplified eDAL  Site-directed mutation    Amplified intein    Amplified sDAL    Amplified sumo3  Amplified ulp1 |

The underlined letters indicate recognition site of specific restriction enzyme.
